# Lymphoid Tissue in Teleost Gills: Variations on a Theme

**DOI:** 10.3390/biology9060127

**Published:** 2020-06-15

**Authors:** Julien Rességuier, Alf S. Dalum, Louis Du Pasquier, Yaqing Zhang, Erling Olaf Koppang, Pierre Boudinot, Geert F. Wiegertjes

**Affiliations:** 1 Department of Biosciences, University of Oslo, Blindernveien 31, 0371 Oslo, Norway; julien.resseguier@ibv.uio.no; 2 Section of Anatomy, Faculty of Veterinary Science, Norwegian University of Life Sciences, Ullevålsveien 72, 0454 Oslo, Norway; alf.seljenes.dalum@gmail.com (A.S.D.); erling.o.koppang@nmbu.no (E.O.K.); 3 Zoology and Evolutionary Biology, University of Basel, 4051 Basel, Switzerland; louis.dupasquier@unibas.ch; 4 Aquaculture and Fisheries Group, Department of Animal Sciences, Wageningen University & Research, 6700 AH Wageningen, The Netherlands; yaqing.zhang@wur.nl; 5 University of Paris-Saclay, INRAE, UVSQ, VIM, 78350 Jouy-en-Josas, France; pierre.boudinot@inrae.fr; 6 Cell Biology and Immunology Group, Department of Animal Sciences, Wageningen University & Research, 6700 AH Wageningen, The Netherlands

**Keywords:** fish, gills, ilt, evolution, zap70

## Abstract

In bony fish, the gill filaments are essential for gas exchanges, but also are vulnerable to infection by water-borne microorganisms. Omnipresent across fish, gill-associated lymphoid tissues (GIALT) regulate interactions with local microbiota and halt infection by pathogens. A special GIALT structure has recently been found in Salmonids, the interbranchial lymphoid tissue (ILT). However, the structural variation of GIALT across bony fish remains largely unknown. Here, we show how this critical zone of interaction evolved across fishes. By labeling a conserved T-cell epitope on tissue sections, we find that several basal groups of teleosts possess typical ILT, while modern teleosts have lymphoepithelium of different shape and size at the base of primary gill filaments. Within Cypriniformes, neither body size variation between two related species, zebrafish and common carp, nor morphotype variation, did have a drastic effect on the structure of ILT. Thereby this study is the first to describe the presence of ILT in zebrafish. The ILT variability across fish orders seems to represent different evolutionary solutions to balancing trade-offs between multiple adaptations of jaws and pharyngeal region, and immune responses. Our data point to a wide structural variation in gill immunity between basal groups and modern teleosts.

## 1. Introduction

The pharyngeal pouches and slits are a fundamental structure shared by all deuterostomes. They were primarily involved in filter feeding. Gills are present within pharyngeal pouches of all vertebrates during embryogenesis, but they fully develop in adult forms of aquatic fishes. This paraphyletic group comprises lampreys and hagfish (Agnathans), cartilaginous fishes (Chondrichthyes) including sharks and rays with a skeleton made of cartilage, and bony fishes (Osteichthyes) with a skeleton made of bone, including the modern bony fishes (Teleostei), [1]. With a vast and complex folded surface, gills are considered crucial for O_2_ uptake, energy turnover and exchange of ions and CO_2_. 

**Figure 1:**
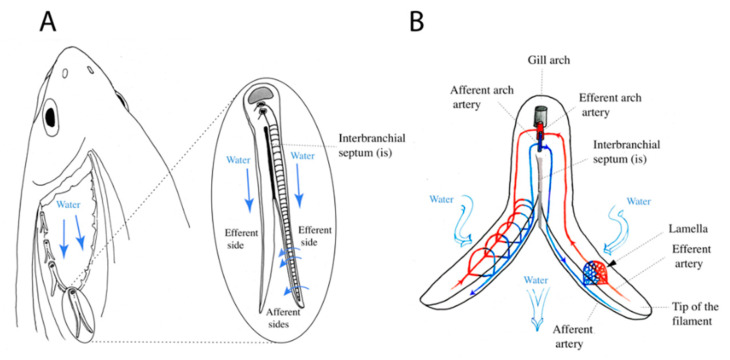
Schematic view of teleost fish gills. (**A**) Individual gill arches each offering support to two rows of gill filaments in alternate positions, forming a sieve-like arrangement. Filaments are supported by interbranchial septa of which the length varies with the species. The septum is most reduced in modern teleosts. (**B**) Schematic representation showing that gill filaments are compartmentalized into an afferent and an efferent side, with bloodstream from the afferent (oxygen-deprived blood) to the efferent side (oxygenated blood) and water circulating in the opposite direction.

Even if they serve the same general function, gills of sharks and bony fishes, which have been separated for a long time in evolution, are built on totally different anatomical planes [2,3]. The gills of bony fish are more efficient respiratory organs than those of cartilaginous fish, and their general organization allowed a reduction of muscular and skeletal elements compared to Chondrichthyes [3,4,5,6]. It stands to reason that the evolution of teleost gills has been driven by respiratory efficiency [7]. All bony fish gills are enclosed in the pharyngeal pouches and lie on cartilaginous bases which are called gill arches. Supported by these arches, the gill respiratory surfaces are always organized in two columns of filaments (also called primary lamellae) that can be separated by an interbranchial septum extending from the gill arch (Figure 1). The gas exchange occurs at secondary structures called lamellae (also called secondary lamellae) that extend from each filament. These lamellae are hollowed flap-shaped structures where an intense blood flow runs in-between supportive cells called pillar cells. A typical branchial epithelium is mainly composed of pavement cells in-between which reside chloride cells (also called mitochondria-rich cells), mucous cells and neuroepithelial cells [3,8]. Filaments are compartmentalized, the external or efferent side of a filament is differentiated from the inner or afferent side and the interlamellar region is positioned in-between. Water flows over the filaments from the efferent aspect to the afferent aspect, in the opposite direction of the blood flow, thus maximizing gas exchange. Within the Teleostei, despite this common anatomical plan, the morphology of the gills also shows large variations between different groups and species. Salmonids and cyprinids have well-developed interbranchial septa, but in percomorphs these are incorporated into the gill arches [9], exemplifying an apparently gradual shortening of the interbranchial septa across fish orders.

Since the water is an excellent environment for pathogens including viruses [10] the mucosal linings covering fish gills appear vital in providing immune defense mechanisms [11]. Gills have thus a diffuse presence of immune cells. In addition, a specialized lymphoepithelial tissue termed the interbranchial lymphoid tissue (ILT) was identified in 2008 in Atlantic salmon (*Salmo salar*) [12]. Subsequent studies in Atlantic salmon and rainbow trout (*Oncorhynchus mykiss*) demonstrated the presence of T lymphocytes embedded in a meshwork of reticulated epithelial cells in ILT. Only few immunoglobulin (Ig)M+ lymphocytes were detected, but the structure appears rich in cells expressing major histocompatibility complex (MHC) class II+ cells and IgT transcripts [12,13,14,15,16,17,18,19,20]. The ILT must be considered as a part of the gill-associated lymphoid tissue (GIALT), which is defined as one of the four main mucosal immune compartments found in bony fish [21]. The salmon ILT was recently found positive for the constitutive expression of CCL19, a cytokine normally associated with expression in lymphoid organs, suggesting that it could be considered as an immune organ in its own right [22]. 

**Figure 2:**
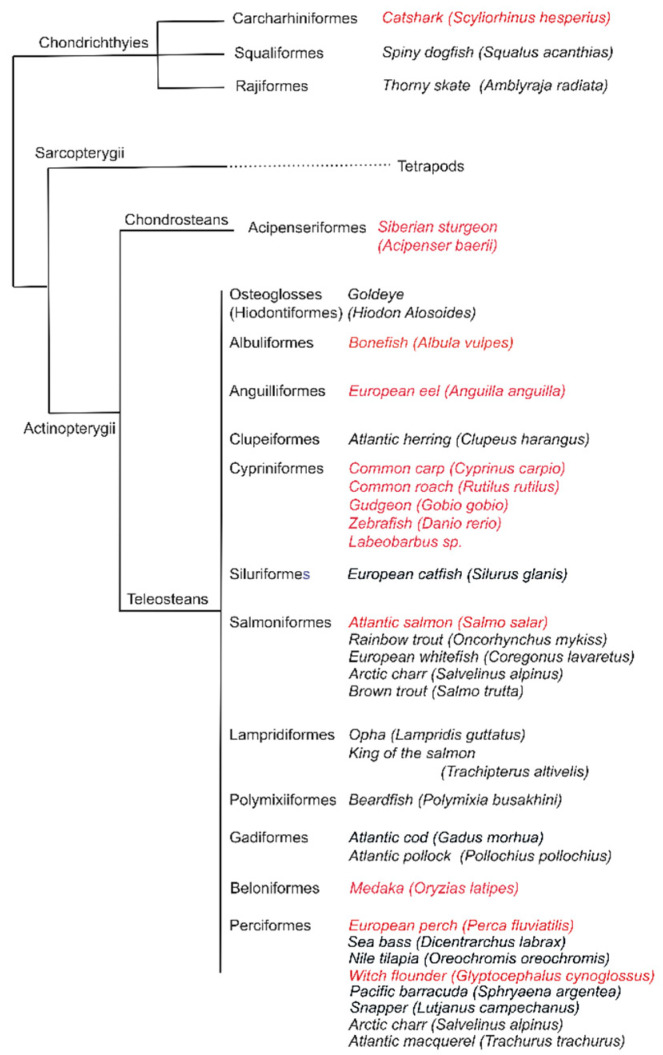
Gill-associated lymphoid tissue (GIALT) is omnipresent across fishes. GIALT was not found in the key group of cartilagenous fishes but was found in representatives of many different key groups across ray finned fishes (*Actinopterygii*), including Chondrosteans. Names in red are key species analyzed in detail in this work. Taxonomy as in [25].

Here, we characterized histologically the structure of ILT among representative orders of bony fish (Figure 2), targeting Zap 70 (Zeta-chain-associated protein kinase 70), a phylogenetically conserved protein, to visualize the presence of both T and NK lymphocytes. We compared the impact of body size on ILT structure, taking advantage of a unique set of samples of African barb representing a rare “species flock” of at least 14 morphotypes with reshaped buccal and pharyngeal regions [23,24]. We also asked whether morphological adaptation to different feeding modes and presumed differences in water flow over the gill region would correlate with morphological variation in the ILT. We discuss possible evolutionary driving forces behind variation in ILT structure between basal and modern teleosts.

## 2. Materials and Methods

### 2.1. Species Investigated

Information about the origin and number of specimens for the different fish species investigated are given in Table S1. The study includes wild-caught species for which only fish with a healthy appearance upon sampling and no obvious histopathological alterations in gill tissue upon histological evaluation were included in the study. Other fish species were laboratory-raised and also here, only healthy fish were sampled. After euthanasia, gill tissue was transferred to 10% neutral buffered formalin and stored at 4 °C before further histological processing, unless indicated otherwise, as also indicated below for confocal imaging.

Neither wild-caught nor laboratory-raised fish were subjected to experimental procedures. This study was performed by well-trained staff authorized to perform fish euthanasia. The details about ethical authorizations are provided in Table S1. The study was carried in contact with local ethics committees and followed strictly European legislation.

### 2.2. Histochemistry

Histochemical staining was performed with Hematoxylin/Eosin (H&E) on formalin-fixed material [16]. Briefly, formalin-fixed tissue was paraffin-embedded and processed for histological analysis using standard procedures. Formalin-fixed, paraffin-embedded gill tissue were then sectioned at 4 μm thickness and mounted on poly-L-lysine-coated slides (Superfrost Plus, Thermo Scientific, Braunschweig, Germany). The slides were incubated at 37 °C for 24 h, then at 58 °C for 24 h, before deparaffinization in xylene. Sections were then rehydrated in graded alcohols to distilled water. Immunohistochemical staining was performed with the anti-Zap-70 rabbit mAb #2705 from Cell Signaling (99F2). The cross-reactivity for the fish Zap-70 protein, a kinase playing a critical role in initiating T-cell responses via the T-cell receptor (TCR) and expressed mainly by T cells and NK cells in human and mice, had been verified for use in common carp [26] and zebrafish [27]. Slides were blocked for 20 min with 0.2% goat normal serum in 5% bovine serum albumin (BSA)/tris-buffered saline (TBS) before incubation with primary antibody of appropriate concentrations diluted in 1% BSA/TBS. Labeled polymer-HRP anti-mouse (Dako EnVison+ System-HRP, Dako, Glostrup, Denmark) was used as the secondary antibody. We examined the omnipresence of GIALT in fishes using the anti-Zap-70 antibody as a universally cross-reactive pan T lymphocyte marker.

### 2.3. Confocal Imaging

Adult zebrafish and medaka (1 year old) were euthanized with an overdose of buffered tricaine, fixed in 4% paraformaldehyde for 24 h at room temperature, cryoprotected in a solution of sucrose 30% for several days, embedded in Tissue-Tek O.C.T. Compound (Sakura Finetek USA, Mountain View, CA, USA), flash-frozen in isopentane, and sectioned using a CM1950 cryostat (Leica, Wetzlar, Germany). The resulting 30 µm cryosections were recovered on a Superfrost Plus slide (Thermo Fischer, Waltham, MA, USA). T and NK lymphocytes were labeled using a rabbit anti-Zap70 monoclonal antibody (clone 99F2–Cell Signaling) at 1:200 with overnight incubation at 4 °C, followed by a 30 min incubation with a cross-adsorbed goat antirabbit secondary antibody (Jackson, Glendora, CA, USA) conjugated to Alexa647 at 1:250. Cryosections were costained with TRITC conjugated phalloidin (Sigma, St. Louis, MO, USA) at 3 U/mL and DAPI (Sigma) at 5 µg/mL during the incubation with the secondary antibody. Slides were mounted with a prolong-glass mounting medium (Thermo Fischer) and cured at room temperature for 24 h. Acquisitions were made with the Zyla camera of a dragonfly spinning disk confocal microscope (Andor, Belfast, UK), with 40 µm pinholes and either a 20×/0.75-dry objective or a 60×/1.4-oil-immersion objective. Acquisitions, stitches and deconvolutions were made using features from the Fusion software. Image analysis was realized using both IMARIS and ImageJ software. Image acquisition and analysis were performed at the NorMIC imaging platform (University of Oslo, Norway).

## 3. Results

### 3.1. Variations of Interbranchial Lymphoid Tissue (ILT) across Representative Fish Orders

**Figure 3:**
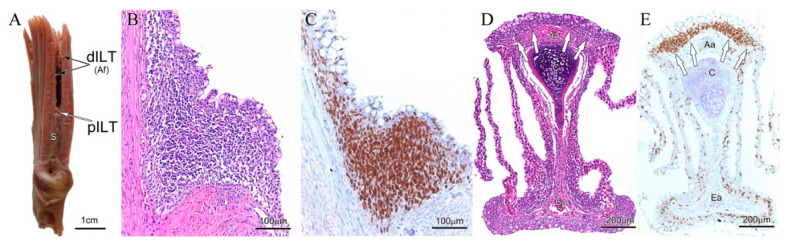
Gill-associated lymphoid tissue (GIALT) or interbranchial lymphoid tissue (ILT) of Atlantic salmon (*Salmo salar*). All images were taken from the second left gill arch. (**A**) Macroscopic image of a transversally dissected gill. The white arrow points to the proximal part of the ILT (pILT) as seen in image (**B**,**C**). Black arrows point to the distal part of the ILT (dILT) as seen in image (**D**,**E**). The interbranchial septum S forms the bottom of the interbranchial cleft. (**B**) Hematoxylin/Eosin (H&E) staining of the ILT showing intraepithelial lymphoid aggregates. (**C**) Zap-70 staining of the corresponding region showing high numbers of Zap-70+ cells (brown). (**D**) H&E staining of a filament (sectioned in the coronary plane with regards to the filament cartilage). White arrows point to the dILT. (**E**) Zap-70 staining of the corresponding region showing high numbers of Zap-70+ cells within the epithelium of the afferent aspect of the filament (i.e., dILT).

The first report of a defined structure in the GIALT of a teleost fish was the discovery of the ILT in the gills of Atlantic salmon (Figure 3A). The ILT of salmon is divided in a proximal portion (pILT) located at the terminal end of the interbranchial septa (i.e., located at the base of interbranchial clefts) and a less distinct and distal portion (dILT) extending along the primary lamellae, developing toward the tip of filaments along the afferent sides. This structure could be visualized by histochemical staining (Figure 3B,D) and appeared to contain a large proportion of lymphocytes. We, therefore, used a monoclonal antibody against the highly conserved Syk family protein tyrosine kinase Zap-70 that is expressed by T cells and NK cells, and known to play a critical role in mediating T-cell activation in response to TCR engagement. The Zap-70 staining allowed us to delineate the ILT of salmon by enhancing the contrast with surrounding tissues and by confirming that the majority of the lymphoid cells are indeed Zap-70+ and thus likely T lymphocytes (Figure 3C,E).

Given the variation in morphology of the gills across bony fish species and the gradual shortening of the interbranchial septa from “basal” to “advanced” fishes, we used the associated interbranchial clefts as an anatomical guide through gill morphology to compare the exact location of the ILT across fishes of different orders. Taking advantage of the cross-reactivity of the anti-Zap70 antibody across a wide taxonomic range, we included in our comparative approach immunohistochemical staining to confirm the presence of lymphocytes in ILT of representative fish species, ranging from shark to modern teleosts.

**Figure 4:**
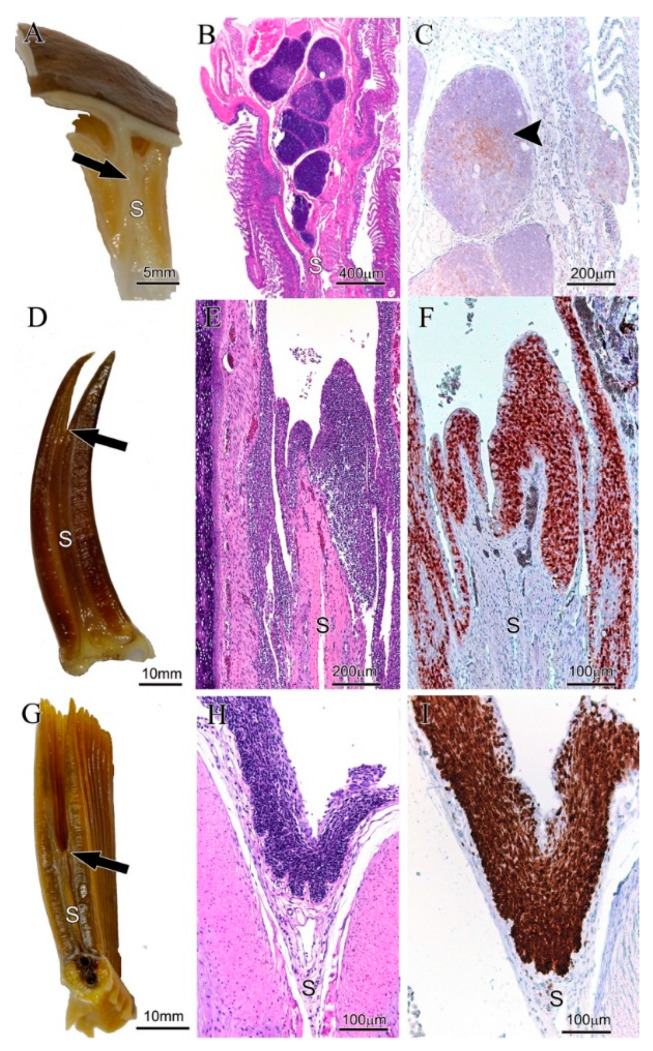
Gill-associated lymphoid tissue (GIALT) of Chondrichthyes and Actinopterygi. All images were taken from the second left gill arch and oriented in the transversal plane of the gill. (**A**–**C**) Catshark (*S. hesperius*). (**A**) Macroscopic image of a dissected gill. Note that the interbranchial septum (S) is continuous with the hypodermis of the outer body surface. The black arrow points to the region as seen in images (**B**,**C**). (**B**) Staining of a transversal sectioned gill arch. Note the thymic tissue with cortex and medulla within the interbranchial septum. (**C**) Zap-70 staining of a thymic lobe. Note the high numbers of Zap-70+ cells (brown) within the medulla (black arrowhead). (**D**–**F**) Siberian sturgeon (*A. baerii*). (**D**) Macroscopic image of a dissected gill. Note the long but discontinued interbranchial septum (S). The black arrow points to the region as seen in images (**E**,**F**). (**E**) H&E staining of the epithelium lining the distal part of the interbranchial septum. (**F**) Zap-70 staining of the corresponding region shown in (**E**). Note the high numbers of Zap-70+ cells (brown) within the epithelium. (**G**–**I**) Bonefish (*Albula vulpes*). (**G**) Macroscopic image of a dissected gill. Note the intermediate length of the interbranchial septum (S). The black arrow points to the region as seen in (**H**,**I**). (**H**) staining of the epithelium lining the distal part of the interbranchial septum. (**I**) Zap-70 staining of the corresponding region shown in (**H**). Note the high numbers of Zap-70+ cells (brown) within the epithelium.

First, we examined the gills of a representative cartilaginous fish species, the catshark (*Scyliorhinus hesperius*). Here, the interbranchial septum connects the gill arches to the outer body wall, thus there are no interbranchial clefts (Figure 4A). We could observe confined areas of Zap-70+ cells in the thymus, which is located in the dorsal region of the gill arches, inside the interbranchial septa (Figure 4B). The antibody staining revealed differentiated cortex- and medulla-like tissues, with a high proportion of the medullary-like cells being Zap-70+ (Figure 4C). The typical cortex- and medulla- labeling pattern is typical of thymus and highlights the cross-reactivity of the anti-Zap-70 antibody with the Zap-70 protein from Chondrichthyes. However, no typical ILT could be detected in catshark by histochemical nor by immunohistochemical examination, despite multiple investigations using different orientations (transversal, sagittal and dorsal).

Second, among Actinopterygians, we studied the gills of a typical Chondrostean, the Siberian sturgeon (*Acipenser baerii*). In this basal bony fish species, the interbranchial septa are not connected to the outer body wall but have free distal ends allowing for short interbranchial clefts (Figure 4D). We could detect, by histochemistry, clear and distinct lymphoid aggregates at the base of the clefts (Figure 4E) that are continuous with the afferent side of the filaments. The presence of these local pILT and dILT aggregates was confirmed by the anti-Zap-70 antibody labeling (Figure 4F) and, as found typical of the ILT from Atlantic salmon, the ILT of sturgeon was always located within the mucosal lining of epithelial cells.

Third, we studied several basal groups of teleosts in which the interbranchial septa and thus also the interbranchial clefts, are reduced to an intermediate length as seen in Atlantic salmon. We started with Elapomorpha (eels and tarpons). The gills of a typical Elapomorpha, the bonefish (*Albula vulpes*) showed lymphoid aggregates containing a high proportion of Zap-70+ cells both, at the base of the interbranchial cleft (pILT) and along the afferent side (dILT) of gill filament epithelium (Figure 4G–I), similar to the ILT of Atlantic salmon. A comparable structure was also found for another well-known elapomorphan species, the European eel (*Anguilla anguilla*). In addition, the groups of the cyprinids (Ostariophysi) also share the proximal and distal structure of ILT, as confirmed for common roach (*Rutilus rutilus*), common carp (*Cyprinus carpio*) and gudgeon (*Gobio gobio*; Supplementary Materials (Figure S1)). Details of the ILT of another key cyprinid species, the zebrafish (*Danio rerio*), will be discussed later in this manuscript.

**Figure 5:**
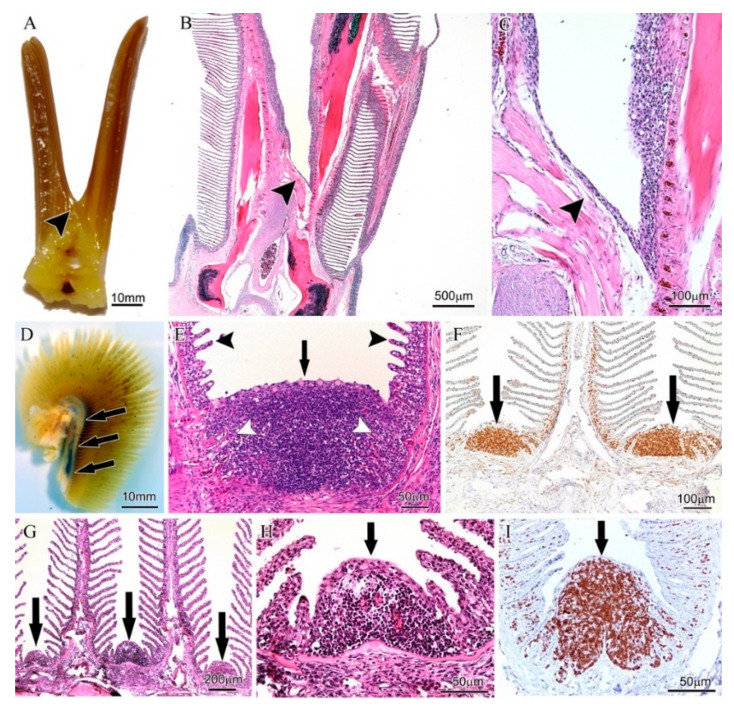
Absence of interbranchial lymphoid tissue (ILT) but the presence of gill-associated lymphoid tissue (GIALT) in the interfilamental space in two Neoteleostian species. (**A**–**F**) European perch (*P. fluviatilis*). (**A**) Macroscopic image of gill in transversal projection. The arrowhead points to the end of the interbranchial cleft as also seen in (**B**,**C**). Note the short or missing interbranchial septum. (**B**,**C**) staining of transversal orientation: no ILT-like structure could be identified in the thin epithelium covering the bottom of the interbranchial cleft. (**D**) Macroscopic image of gill in a lateral projection. (**E**,**F**) Sagittal orientation: Extensive intraepithelial lymphoid aggregates between the proximal part of the filaments at their attachment to the gill arch. The black arrows in (**D**) show the orientation in the histological sections (**E**,**F**), pointing to the transition between the gill arch and the filament where the intraepithelial lymphoid aggregates are situated. (**E**) H&E staining; black arrowheads point to free lamellae, while white arrowheads point to lamellar structures embedded within the intraepithelial lymphoid aggregate. (**F**) Immunohistochemical staining using the anti-Zap-70 antibody to indicate Zap-70+ cells (brown). All images were taken from the second left gill arch. (**G**–**I**) Witch flounder (*G. cynoglossus*). Sagittal orientation: black arrows show orientation in the histological section pointing to the transition between the gill arch and the filament where intraepithelial lymphoid aggregates are situated. (**G**–**H**) Extensive intraepithelial lymphoid aggregates between the proximal part of the filament at their attachment to the gill arch. (**I**) Immunohistochemical staining using the anti-Zap-70 antibody to indicate Zap-70+ cells (brown). All images were taken from a second left gill arch.

Fourth, among Neoteleostei, we first focused on a representative spiny-rayed fish (Acanthopterygii), the European perch (*Perca fluviatilis*). Perch gills typically show very short, quasi-absent interbranchial septa and thus deep interbranchial clefts (Figure 5A). This allows for a wider angle between gill filaments, offering more space at the base of the clefts. In strong contrast to basal groups of teleosts, aggregates of Zap-70+ cells were found only at the base and afferent side of filaments and could not be considered ILT-like structures. Similar observations were obtained from another spiny-rayed fish species; the Witch flounder (*Glyptocephalus cynoglossus*). For these spiny-rayed fish species without apparent ILT-like structures, investigation of gills in the sagittal plane (see Figure 5D) showed the presence of Zap-70+ lymphoid aggregates within the branchial epithelium, as denoted by the presence of mucous cells, between the proximal part of the filaments at their attachments to the gill arch (Figure 5E,F for perch); (Figure 5G–I for flounder). To our knowledge, such lymphoid aggregates have not been described previously. We consider these aggregates part of the GIALT.

### 3.2. Body Size Variation Does Not Impose Drastic Restructuring of the ILT

The size of an organism has an important impact on its physiology and morphology, size reduction often leading to a loss of tissue complexity. In the immune system, the absolute number of lymphocytes has important consequences on the diversity and structure of B- and T-cell repertoires [28]. Intuitively, one may, therefore, expect that body size could significantly influence the relative size and structure of ILT. 

**Figure 6:**
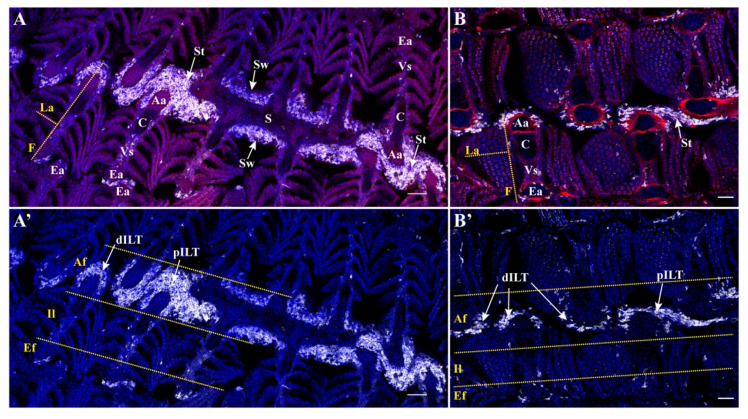
Gill-associated lymphoid tissues (GIALT) of zebrafish (*D. rerio*). Spinning disk confocal deconvolved images of adult zebrafish gills coronal sections across and above the interbranchial septum (**A**,**B’**). (**A**,**B’**) represent 10 µm maximum intensity projection (MIP) from a multifields stitch (20× objective) displaying phalloidin/DAPI/zap70 labeling for (**A**,**B**) and DAPI/zap70 labeling for (**A’**,**B’**). Images were acquired from 30 µm thick whole-organism cryosections, while tissues structures are highlighted with phalloidin (F-actin, red) and DAPI (Nuclei, blue) stainings in order to examine the distribution of Zap-70+ cells (white). (**A**,**A’**). Zap-70+ cells are mainly concentrated within the afferent aspect of the filaments. Note the high number of Zap-70+ cells within the septum wall, which then assemble into the proximal ILT (pILT) at the uppermost layer of the interbranchial septum. (**B**,**B’**). Zap-70+ cells then extend upward from the top of the interbranchial septum to form the distal ILT (dILT) along the afferent aspect of filaments. In addition to the structured lymphoid tissue observed, Zap-70+ cells are also distributed in a diffuse manner among the interlamellar region and the efferent aspect of filaments (**A’**,**B’**). F, Filament; La, Lamellae; S, Septum; St, Septum top; Sw, Septum wall; Af, Afferent aspect; Il, interlamellar region; Ef, Efferent aspect; Aa, Afferent artery; Vs, Venous sinus; Ea, Efferent artery; C, Cartilage. Scale bar: 50 µm (**A**,**A’**) and 30 µm (**B**,**B’**).

We compared the ILT and its associated expression of Zap-70 described for common carp (see Figure S1) with that of zebrafish (*D. rerio*), a well-known species widely used in developmental biology, human-disease modeling and drug screening. Both are closely-related cyprinid species but an adult zebrafish, weighing less than one gram, is thousands of times smaller than an adult carp. Remarkably, the gills of zebrafish showed a high number of Zap-70+ cells located at the base of the interbranchial clefts (Figure 6) and along the afferent side of the filaments, typical of ILT of common carp. As in carp, scattered Zap-70+ lymphoid cells are also found on the efferent side and interlamellar region. Overall, the ILT of these two cyprinid species of different sizes is elegantly structured in a highly comparable manner.

**Figure 7:**
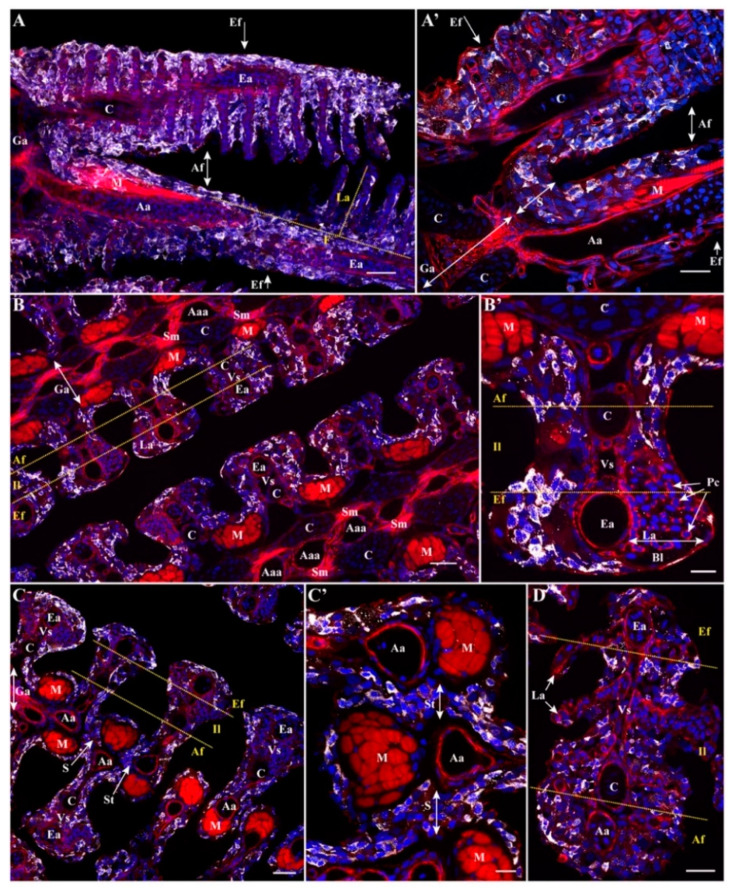
Gill-associated lymphoid tissues (GIALT) of medaka (*O. latipes*). Spinning disk confocal deconvolved images of adult medaka gills paratransversally sectioned (**A**,**A’**), coronally sectioned beneath the septum (**B**,**B’**), coronally sectioned across the septum (**C**,**C’**) and coronally sectioned above the septum (**D**). (**A**) is a 10 µm MIP (20× objective). (**A’**) is a 1 µm MIP (60× objective). (**B**) is a 1 µm MIP from a 6 fields of view stitch (60× objective) showing two successive gill arches. (**B’**) is a reoriented and zoomed area from (**B**). (**C**) is a 1 µm MIP from a multifields stitch (60× objective). (**C’**) is a reoriented and zoomed area of (**C**). (**D**) is a 1 µm MIP (60× objective). Images were acquired from 30 µm thick whole-organism cryosections, while tissues structures are highlighted with phalloidin (F-actin, red) and DAPI (Nuclei, blue) stainings in order to examine the distribution of Zap-70+ cells (white). (**A**) Note the almost inexistent interbranchial septum (S). Numerous Zap-70+ cells are seen scattered among gill filaments, lamellae and septa without any structural organization. (**A’**) Further magnification illustrating the diffuse presence of the Zap-70+ cells within the very short interbranchial septum and both the efferent and the afferent aspects of the filaments. The diffuse distribution of Zap-70+ cells is consistent all along the filaments, with cells present among the afferent aspect, the efferent aspect and the interlamellar region (delimitated by the yellow dotted lines) underneath the septum (**B**,**B’**), at the septum (**C**,**C’**) and above the septum (**D**). Ga, Gill arch; F, Filament; La, Lamellae; S, Septum; St, Septum top; Af, Afferent aspect; Il, interlamellar region; Ef, Efferent aspect; Aaa, Afferent arch artery; Aa, Afferent artery; Vs, Venous sinus; Ea, Efferent artery; C, Cartilage; M, skeletal muscles; Sm, Smooth muscle; Pc, Pillar cells; Bl, Blood vessel lumen. Scale bar: 30 µm (**A**–**C**), 20 µm (**A’**,**D**) and 10 µm (**B’**,**C’**).

We also compared the expression of Zap-70 we described for the spiny-rayed fishes perch and flounder (Figure 5) with that of medaka (*Oryzias latipes*), a well-known species widely used as a model for toxicology and developmental biology. As above, adult medaka are thousands of times smaller than adult perch and flounder. These species are all Percomorphs (Acanthopterygii) with interbranchial septa that have been reduced to a minimum. In the gills of medaka, like the two other species no typical ILT could be identified. Only scattered Zap-70+ lymphoid cells could be observed (Figure 7).

### 3.3. Adaptation of Jaw Morphology to Different Feeding Modes Does Not Impose Drastic Restructuring the ILT

**Figure 8:**
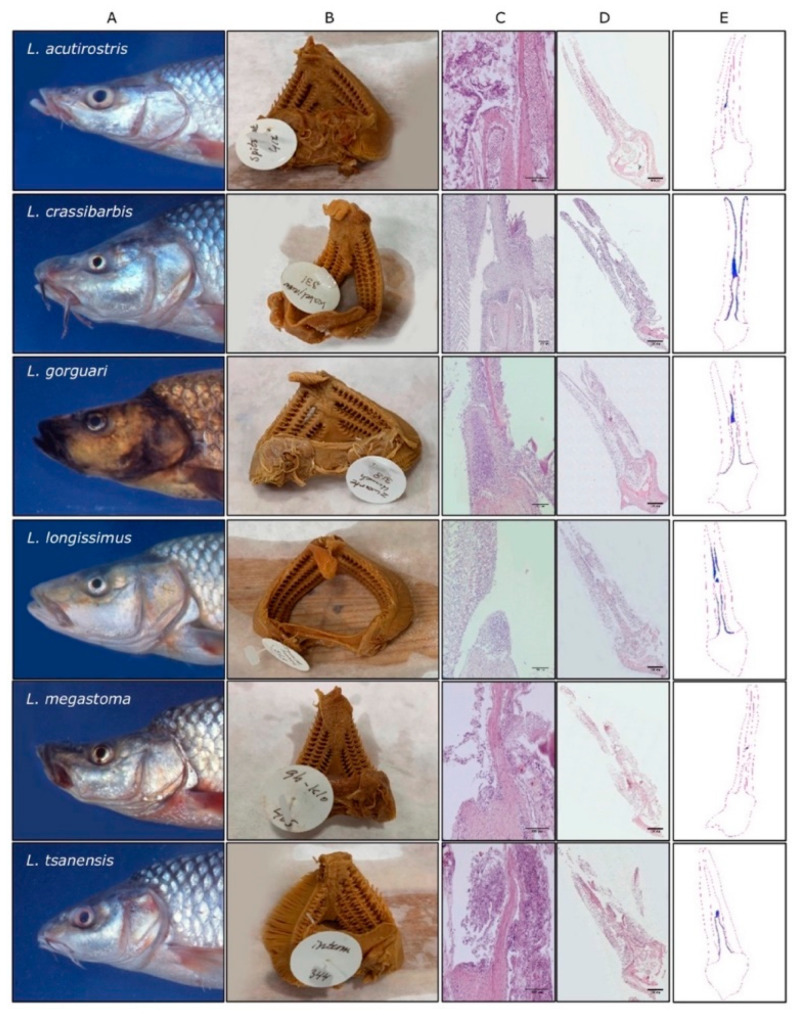
Heads of 6 endemic *Labeobarbus* species flock in Lake Tana (column (**A**)) and their isolated gills (column (**B**)) after 24-h fixation in formalin and followed by 26-year storage in 70% alcohol [24], and a transverse view of lymphoid tissue (ILT) in HE-stained gills (Scale bar is 100 μm, column (**C**)), transverse view of HE-stained gills (Scale bar is 500 μm, column (**D**)), transverse drawing of gill morphology (column (**E**)). The transverse drawings of gill morphology are based on scanned HE-stained gills, and lymphocyte-like cell accumulations are in blue (column (**E**)).

Adaptive radiation in unstable environments leads to fast morphological change. Classical examples are provided by the beak of Darwin’s finches [29] and by the body shape and armor phenotype of stickleback [30]. The structure of fish jaws also evolves under strong selection: the upper jaw of several cichlid species from Lake Victoria and barbels from Lake Tana showed very fast divergent adaptive changes upon food resource fluctuations [31], potentially affecting the cranial structure and gill arches. *Barbus intermedius* is a cyprinid fish species from Lake Tana (Ethiopia) and a hexaploid taxon that formed a unique ‘species flock’ consisting of at least 14 morphotypes. Feeding specializations introduced rapid reshaping of buccal and pharyngeal regions [23,24] with presumed differences in water flow over the gill region of different morphotypes. Access to long-term conserved samples of highly diverse morphotypes prompted us to examine the diversity in ILT in this unique collection. Even in the gills of highly divergent Barbus morphotypes (Figure 8), although difficult to evaluate in detail because tissues clearly suffered from the long conservation period, we did not observe significant modifications of ILT as found typical of closely-related cyprinids such as common carp and zebrafish. Altogether, this dataset suggests an overall conserved ILT structure along cyprinids.

## 4. Discussion

Defined lymphoid structures associated with fish gills have first been discovered in Atlantic salmon only a few years ago and were named interbranchial lymphoid tissue (ILT). In this species, gill filaments are joined about half their length by supportive septa, placing the ILT (i.e., proximal ILT (pILT)) on its distal end, at the base of the interbranchial clefts. This GIALT structure extends to include lymphoid aggregates at the afferent sides of the gill filaments (i.e., distal ILT (dILT)). Given that this structure has been described primarily in Atlantic salmon and rainbow trout, here we investigated to what extent ILT has evolved between basal fish groups and modern teleosts. Given that both, body size and morphology of the jaw and pharyngeal region can be highly variable across teleosts, potentially modifying the location of immune cells in gills, we also investigated if this would influence the structure of ILT across teleosts. We made use of the fact that Zap-70 is a phylogenetically conserved protein, which helped to visualize the location of T lymphocytes within gills across key orders of bony fish. While GIALT structures are omnipresent across Actinopterygians, our data show that ILT placed at the base of the interbranchial clefts is variable across bony fishes. Its absence in some groups that lack interbranchial septa suggests the presence of a close coevolution of these structures.

In this study we used the gradual shortening of the interbranchial septa from “basal” to “advanced” fishes as an anatomical guide through gill morphology to compare the exact location of structured elements of ILT across fishes of different orders. In sharks, the interbranchial septa connect the gill arches to the outer body wall. Hence there are no interbranchial clefts. In shark gills we could not detect a definite ILT in-between the filaments. Percomorpha such as perch and flounder have very short, quasi-absent interbranchial septa and thus have deep interbranchial clefts. It proved difficult to define the lymphoid and largely Zap-70+ aggregates found at the base and afferent side of the filaments of modern teleosts such as perch and flounder, as typical ILT. Medaka does not seem to possess at all the structured lymphoid aggregates found in perch and flounder, which underscores the existence of a large variation in GIALT among fishes, in particular among Percomorpha. 

In many, although not in all bony fish species we investigated, we did discover the presence of ILT at the base of interbranchial clefts and at the afferent side of filaments. When exposed to turbid water gills are in contact with a tremendous amount of potentially contaminating particles. Food particles and potential pathogens pass through the gills and water flows along GIALT. During respiration the water flow from the efferent (i.e., leading) to the afferent (i.e., trailing) edges of each side to finally meets in the interbranchial clefts, which, therefore, have been suggested as sites of extensive water-mixing [6]. It is easy to imagine that exposure to high numbers of water-borne particles including potential pathogens at these sites could be a driving force for the development and location of mucosal structures such as ILT at the bottom of interbranchial clefts. Even if gills developed initially for ion regulation rather than gas exchange from water [32], it is likely that the base of interbranchial clefts allows for structures such as ILT to be strategically located because of water flow. Indeed, the development of lymphoid tissues in the respiratory pathways of higher vertebrates such as nasopharynx-associated lymphoid tissue, bronchus-associated lymphoid tissue and tonsils appears highly dependent on environmental influences [33,34,35] and location typically is associated with branching-points of increased interaction between the respiratory medium and the mucosal lining [36]. In gills of fishes with short interbranchial septa, there is less need for upwelling of water along the septal water channels [37], supportive of our observations that ILT placement at the base of the interbranchial clefts is not only variable across bony fishes but could parallel developmental shortening of the interbranchial septa.

The environment (temperature, season, salinity, etc.) might have an effect on gill morphology. We never detected alteration of gill integrity such as fusion of lamellae, hyperplasia of epithelia (likely lymphocyte proliferation), tissue damage or change in the cell composition of epithelia, which could suggest the effect of pollutants. Importantly, our analysis of the morphology of ILT in numerous fish species from totally different biotopes (different salinity, different aquatic environment, different regions) argues against these environments having an overruling effect on the presence of ILT. The absence of true ILT in species like flounder and perch was observed from totally different environments, which further supports our conclusions. Finally, even in fish grown in clean facilities (zebrafish, medaka, eel, carp) we did not notice major discrepancies compared to specimens from the wild (e.g., *Labeobarbus*).

## 5. Conclusions

Our survey across a number of species from diverse taxonomic groups leads to the notion that neither individual size nor anatomy of fish jaws drastically affects the structure of GIALT. Comparison of a number of cyprinids of various sizes (carp, roach, gudgeon, zebrafish) and with various feeding modes and different mouth/head shapes (Barbus species flock) did not reveal drastic differences and we found a comparable fundamental structure of ILT-like structures. Yet, we did notice a major transition that seems to have occurred at the basis of Percomorphs, where the septum virtually disappeared, and gill filaments draw a wide-angle leaving fully accessible the base of the cleft on the gill arch. Our data suggest the “taxonomic rule” determines the ILT structure.

## Supplementary Materials

The following are available online at https://www.mdpi.com/2079-7737/9/6/127/s1, Table S1. Origin of samples and numbers of individuals; Figure S1: Gill-associated lymphoid tissue (GIALT) or interbranchial lymphoid tissue (ILT) of various teleost species.
